# Predicting Master’s students’ academic performance: an empirical study in Germany

**DOI:** 10.1186/s40561-022-00220-y

**Published:** 2022-12-23

**Authors:** Sarah Alturki, Lea Cohausz, Heiner Stuckenschmidt

**Affiliations:** Data and Web Science Group, Faculty of Business Informatics and Mathematics, University of Mannheim, Mannheim, Germany

**Keywords:** Educational data mining (EDM), Higher education, Students’ dropout, Academic achievement, SMOTE

## Abstract

The tremendous growth in electronic educational data creates the need to have meaningful information extracted from it. Educational Data Mining (EDM) is an exciting research area that can reveal valuable knowledge from educational databases. This knowledge can be used for many purposes, including identifying dropouts or weak students who need special attention and discovering extraordinary students who can be offered lifetime opportunities. Although former studies in EDM used an extensive range of features for predicting students’ academic achievement (in terms of (i) achieved grades or (ii) passing and failing), those features are sometimes not obtainable for practical usage, and therefore, the prediction models are not feasible for employment. This study uses data mining (DM) algorithms to predict the academic performance of master’ s students by using a non-extensive data set and including only the features that are easy to collect at the beginning of a studying program. To perform this study, we have collected over 700 students' records from 2010 to 2018 from the Faculty of Business Informatics and Mathematics at the University of Mannheim in Germany. Those records include demographics and post-enrollment features such as semester grades. The empirical results show the following: (i) the most significant features for predicting students' academic achievements are the students’ grades in each semester (importance rate between 14 and 36%), followed by the distance from students’ accommodation to university (importance rate between 6 and 18%) and culture (importance rate between 7 and 17%). On the other hand, gender, age, the numbers of failed courses, and the number of registered and unregistered exams per semester are less significant for the predictions. (ii) As expected, predictions performed after the second semester is more accurate than those performed after the first semester. (iii) Unsurprisingly, models that predict two classes yield better results than those that predict three. (iv) Random Forest classifier performs the best in all prediction models (0.77–0.94 accuracy), and using oversampling methods to deal with imbalanced data can significantly improve the performance of DM methods. For future work, we recommend testing the predictive models on other master programs and a larger datasets. Furthermore, we recommend investigating other oversampling approaches.

## Introduction

As the world shifts towards a global economy, individuals and nations have realized that higher levels of education are essential for competitiveness and success. Therefore, pursuing a master’s degree is considered a well-established postgraduate qualification in higher education. It supports building students’ current abilities and help them acquire new skills related to a particular profession. In light of the increasing interest in master's degrees worldwide, failure or drop-out rates are also high. Observation of dropout can take place either from the position of the university institution, which loses a student, or from the viewpoint of the student, who abandons the pursuit of his/her degree. A university dropout is considered a form of academic failure and the necessity of eliminating it is justified by at least four reasons, (i) economic, (ii) social, (iii) individual, and (iv) pedagogical (Staiculescu, 2018). Therefore, many countries have programs in place that promote opportunities to increase the number of highly qualified people for the knowledge society and economy (Kehm et al., 2019). For instance, several projects in Germany have targeted reducing the number of student dropouts as a strategy to enhance the number of professionals who can join the labor market (Mouton et al., 2020). However, the drop-out rate for master’s programs reached 15% for German students and 28% for international students (Kercher, 2018).

Although retention rates of master's students have been widely documented, there are no solid models for predicting students’ success (Rotem et al., 2020). To minimize the wasting of financial and human resources caused by failure or dropouts, it is vital to build models that can predict atrition at the earliest stage possible. Implementing DM methods to educational data is called Educational Data Mining (EDM) (Baker & Yacef, 2009). EDM is a recent research field gaining popularity because of its high potential for improving educational institutions (Baradwaj & Pal, 2011). It concerns developing methods that discover knowledge from educational environment data (Han et al., 2011). It is built from various fields, including data mining (DM) and machine learning, information visualization, computational modeling, psychometrics, and other areas of statistics (Romero & Ventura, 2007). It also concerns social science as it deals with students’ behavior from social and cultural aspects. EDM methods can provide educators and students with valuable insights into the education process, resulting in suitable actions and decisions that improve academic success (Kotsiantis, 2009). The power of EDM can bring numerous advantages. It can help attract, retain, and motivate students’ success. Moreover, it can assist instructors in tracking students’ progress to improve their teaching methods. It can also help students in the process of course selection and educational management. It can also provide students with valuable feedback, offer recommendations, support personalized learning, allocate scholarships, and discover potential Ph.D. candidates.

There are five main methods of EDM (Baker et al., 2011). Those methods are: (i) relationship mining, (ii) prediction, (iii) clustering (iv) distillation of data for human judgment and (v) discovery with models. In our study, we focus on the first two types. To be more precise, there are three types of predictions in higher education: (i) predicting students' academic performance or GPA at a degree level, (ii) predicting students' failure or drop out of a degree, and (iii) predicting students' results in particular courses (Alturki et al., 2020). In this study, we perform the first and second types. Our primary research questions are:

- *R1* Is it possible to accurately predict the final academic achievement of master’s students?
- *R1* What attributes have the largest effect on the prediction of students’ academic achievement?

After explaining EDM and introducing our research questions, the rest of the paper is organized as follows: the next section presents related work on predictions performed in higher education. Following that, our research methodology is explained. Afterward, we provide details of the experimental results and discussion. Then, we outline the limitations of this study. Finally, we conclude with a summary of the study's primary outcomes and outline future lines of research.

## Literature review on predicting students’ academic performance

According to Rotem et al. (2020), the conducted research regarding students' dropout and postponement at the undergraduate level is more than at the postgraduate level, and no solid predictive models are to be found for postgraduates. For instance, Alemu Yehuala (2015), Aulck et al. (2017), Daud et al. (2017), Pradeep and Thomas, (2015) and Shakeel and Butt (2015) predicted bachelor's degree drop out, Alturki and Alturki (2021), Pal and Pal (2013), Sembiring et al. (2011), Yadav et al. (2011) and Yadav and Pal (2012) predicted bachelor's students' academic achievement at a degree level, and Badr et al. (2016), Huang and Fang (2013), Kovačić (2010) and Osmanbegović et al. (2012) predicted bachelor's students' academic achievement at a course level. The above-mentioned researchers mostly used decision tree algorithms to perform their predictions. They used different type of features. However, gender, age, GPA, income, employment status, and attendance are the most used features.

Based on Nadeem et al., (2021), postgraduate students also face challenges leading to dropout or delay in the program that has remained unexplored. Table 1 summarizes some of the few academic prediction studies that have been performed on a master’s degree level. It compares the different prediction types, the used features in each study, the used algorithms, and the achieved results.

**Table 1:** Academic predictions performed on a master’s degree level

Authors	Prediction type	Used features	Used algorithms	Results
Nghe et al. (2007)	Academic performance	Demographics (marital status, Gross National Income, age, gender), Pre-enrolment features (academic institute, entry GPA, English proficiency, TOEFL^a^ score etc.)	C4.5; Bayesian Networks	They found that C4.5 performs better then Bayesian Networks. They also found that the prediction accuracy of 2 classes (pass and fail) is much higher than that of 3 or 4 classes. Their results also show that the highest accuracy is achieved for the largest class (“Very Good” students)
Yadav et al. (2011)	Academic performance	Post-enrolment features (attendance, test grade, seminar grade, assignment grade, and lab work)	CART; ID3; C4.5	They found that CART produced the best accuracy (56.25%) followed by ID3 (52.08%), then C4.5 (45.83%)
Zimmermann et al. (2011)	Academic performance	Pre-enrolment features (undergraduate achievements)	Random Forest	They found that third year bachelor's achievements are more predictive than the first-year grades in predicting the master’s students’ GPA
Zewotir et al. (2015)	Time to graduate or dropout	Demographics (race, gender, age, and financial aids); Post-enrolment features (major, and term records)	Survival analysis	They found that age and financial aids affect the prediction. However, gender does not. They also found that race has no effect on predicting dropouts. However, it influences the time it took to graduate. Moreover, students’ studying duration is affected by their major
Badr et al. (2016)	Course grade	Post-enrollment features (English grade, and course grade)	CBA rule-generation	They found that CBA rule-generation produced an acceptable accuracy (between 62.75% to 67.33%)
Calisir et al. (2016)	Academic performance	Demographics (gender and employment status)Pre-enrolment features (ALES score^a^, English proficiency exam score, and undergraduate GPA)	Logistic Regression	They found that ALES score, English proficiency exam score, undergraduate GPA, and employment status are the features important for performing their predictions
Jeno et al. (2018)	Academic performance & drop out of a degree	Post-enrollment features (controlled motivation, autonomous motivation competence, need-supportive teachers, and students' intrinsic aspirations)	Regression	They found that "autonomous motivation" and "perceived competence" positively predict academic achievement and negatively predict dropout intentions
Abu Zohair (2019)	Course grade	Demographics (age) Pre-enrolment features (bachelor’s degree type and bachelor’s degree GPA) Post-enrollment features (course grades and instructors’ names)	Neural Networks; Naive Base; Support Vector Machine; K-nearest Neighbor; Linear Discriminant Analysis	They found that Support Vector Machine and Linear Discriminant Analysis perform the best in comparison with the rest of the classifiers
Rotem et al. (2020)	Drop out of a degree	Demographics (Background features) Post-enrollment features (academic performance)	Logistic Regression	They found that using Logistic Regression can accurately predict academic failure and academic performance features predict dropping out better than background features
Zhao et al. (2020)	Academic performance	Demographics (age, marital status, gender, citizenship, Pre-enrolment features (GRE grade^b^, TOEFL grade, months since bachelor’s degree, previous GPA, previous major, previous school rank, previous school country, and previous school language)	Decision Tree; Support Vector Machine; Neural Networks; Naïve Base; K-nearest Neighbor; Ensemble Learner L; Random Forest; Logistic Regression	They found that Random Forest and the Ensemble Learner L achieved the two best overall predictive accuracy

^a^Test of English as a Foreign Language

^b^A taxonomic examination for postgraduate students to enroll in Turkish universities

From the studies reviewed in Table 1, most academic prediction studies have been performed on a degree level. On the other hand, only one study by Abu Zohair (2019) was performed on a course level. Moreover, we can notice that predicting the time to complete or not complete a degree is still not common, especially on a master’s level, as we reviewed only one study by Zewotir et al. (2015). When looking at the type of input data that researchers use, we notice that there is a variation from personal and family related features to income and financial aids features. However, post-enrollment features, such as achieved grades are the most common. It can also be seen that some researchers used attributes that are difficult to acquire, such as personality related features by Jeno et al. (2018) and attendance by Yadav et al. (2011). Despite this, other, easily obtainable attributes that could have been relevant have not been included in the previous studies. For instance, culture's influence on academic predictions was not examined in any of the viewed studies. When comparing students' behavior based on their culture, it is essential to know that cultures are typically divided into collectivist and individualist (Moore et al., 2018). Individualist cultures (e.g., people from the USA, Australia, and Europe) impress personal achievement regardless of the expense of group goals, resulting in a strong sense of competition, while collectivist cultures (e.g., people from Pakistan, India, and the Middle East) impress family and team goals over individual requirements (Kim, 1995). Such differences can have a significant impact on students' overall performance. Therefore, it is vital to investigate the impact of culture on academic predictions. Based on the Deutsche Akademische Austauschdienst (DAAD) (Kercher, 2018), the number of international students at German higher education institutions has increased significantly over the past few years, especially in master's courses. This creates the need to investigate the impact of culture in our study. Moreover, none of the studies performed at a master's degree level used the distance from the students' accommodations to the university as a feature for performing academic predictions. Distance increases the financial and personal costs associated with attending classes, which restricts individual choices and leads to low participation rates (Vieira et al., 2018). Consequently, students who live far from campus have a higher likelihood of failing or dropping out. Therefore, in our study, we choose to include distance as one of the predictive features as we believe that it can have an impact on the academic predictions.

## Methodology

This section of the paper presents an overview of the performed study, the type of collected data, the data analysis, the used DM algorithms, and evaluation methods.

### Data collection

The data set of over 700 students used in this study has been obtained from the Business Informatics and Mathematics faculty at the University of Mannheim from 2010 till 2018. It should be noted that we have followed the European data protection regulations for performing this study and all the collected records have been anonymized prior to working on them. For the purpose of ensuring the reliability of the data, we excluded those students who had not completed their degrees prior to the Covid-19 pandemic. Among the reasons for the exclusion are the drastic changes in the nature of examinations and learning styles, e.g., online exams.

The Business Informatics master’s program's intended duration is four semesters (two years) with approx. 120 European Credit Transfer System (ECTS). However, it usually takes up to six semesters. Students in German universities have the option to register or unregister for course examinations each semester. Therefore, it is common that students postpone an examination for the next semester or the one after. The number of registered exams represents the amount of studying load, i.e., the more registered exams, the more the load is on the student. For our prediction study, we intend to select only easy-to-collect attributes that can be collected from any university database, as shown in Table 2. We have used a combination of demographics and post-enrollment features.

**Table 2:** Description of the collected data that is used to predict the academic achievement

Feature	Description	Type	Value
Academic_status	Whether the student completed the master’s degree or not	Nominal	Completed, and Not_completed
Academic_grade	Student's final achieved grade	Nominal	Above average, Average, and Below average
Gender	Student's gender	Nominal	Male, Female
Enrollment_age	Student's age at the time of enrollment	Numeric	21–38
Culture	Student's culture	Nominal	Collectivistic, and Individualistic
Distance	Distance from accommodation to the university campus	Numeric	≥ 1 km
Grade_sem1	Student’s average grade in the 1st academic semester	Numeric	1 – 5
Grade_sem2	Student's average grade in the 2nd academic semester	Numeric	1 – 5
F_sem1	Number of failed courses in the 1st semester	Numeric	≥ 0
F_sem2	The Number of failed courses in the 2nd academic year	Numeric	≥ 0
Unregistered_exams1	The number of courses that have been taken in the 1st semester, however, have not taken the exam	Numeric	≥ 0
Unregistered_exams2	The number of courses that have been studied in the 2nd semester, however, did not take the exam	Numeric	≥ 0
Registered_exams1	The number of courses that have been examined in the 1st semester	Numeric	≥ 0
Registered_exams2	The number of courses that have been examined in the 2nd semester	Numeric	≥ 0

### Data analysis

**Fig. 1:**
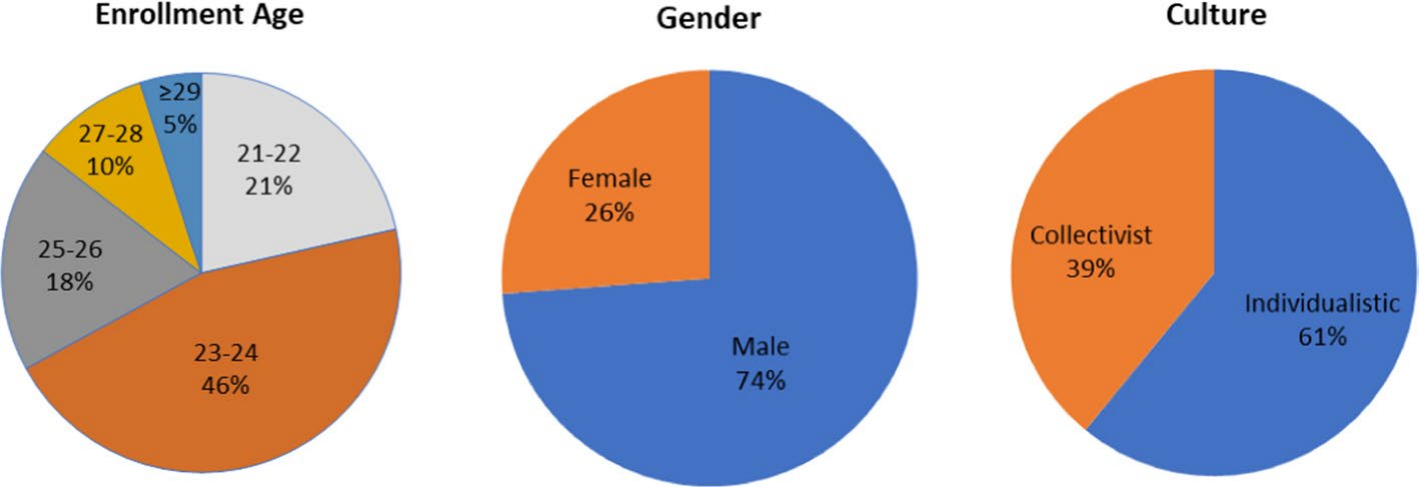
Students' demographical features

**Fig. 2:**
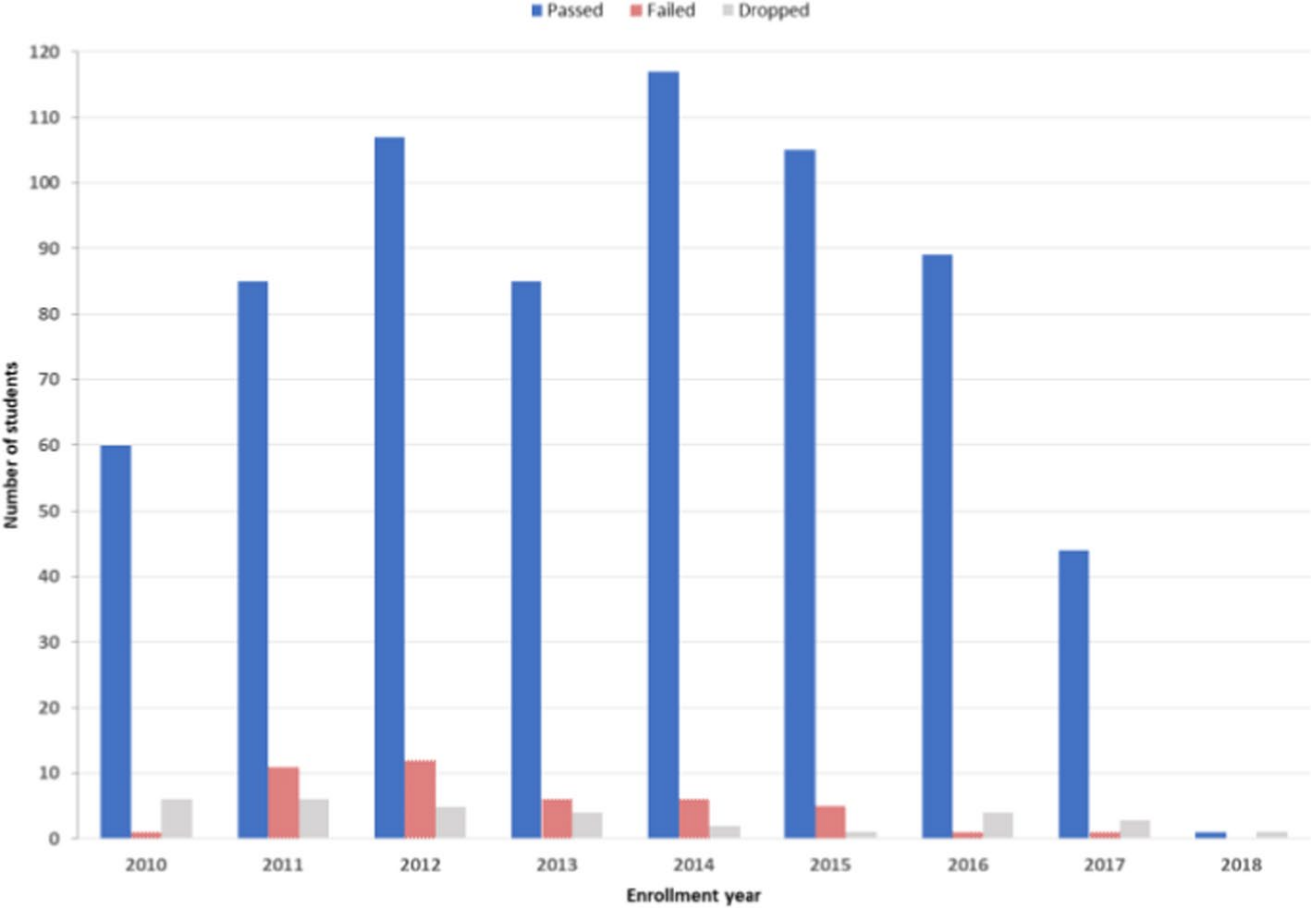
Students' Academic status

**Fig. 3:**
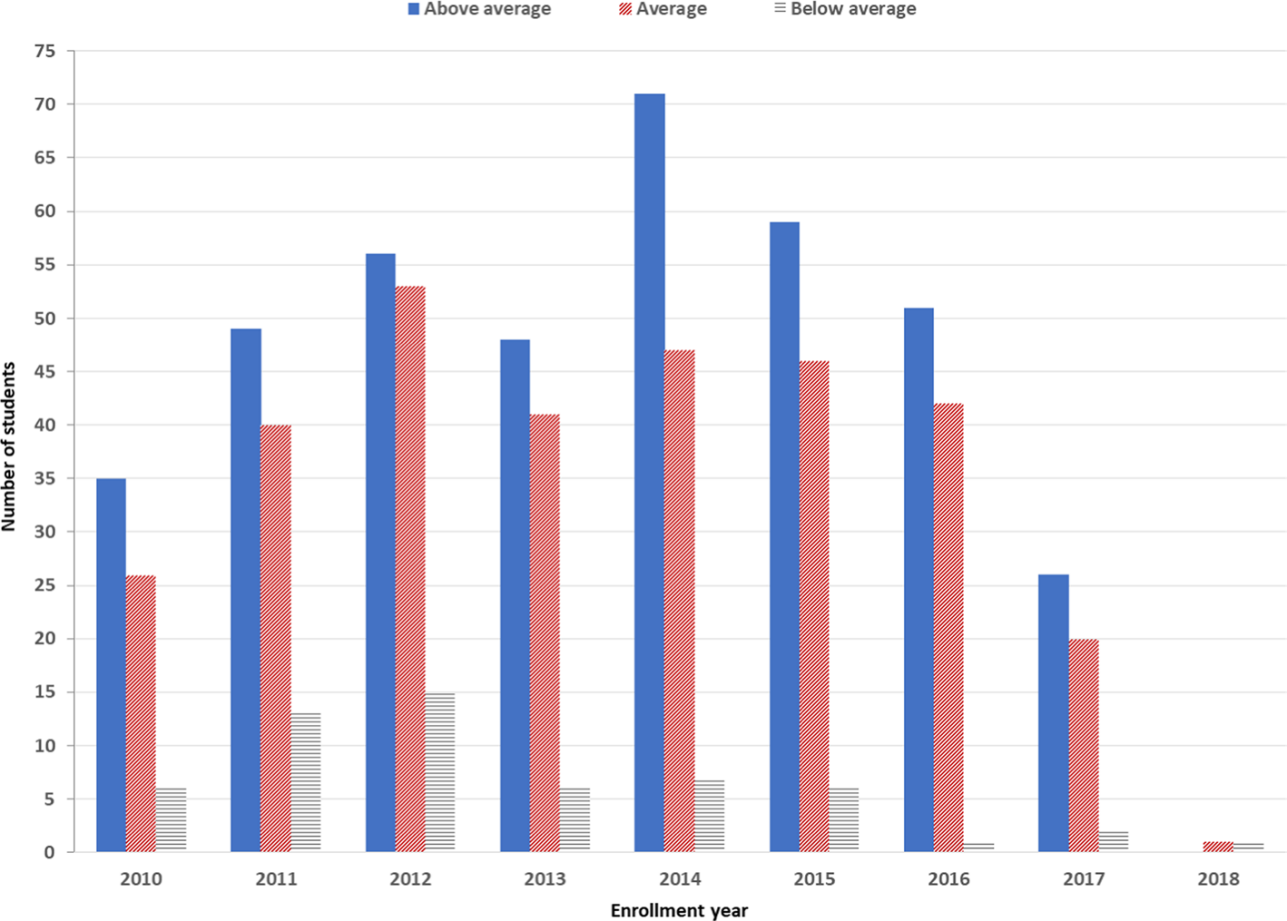
Students’ Academic grade

Before performing the academic achievement predictions, it is essential to analyze the dataset at hand. As shown in Fig. 1, the number of male students significantly exceeds the number of females. Furthermore, the number of students coming from individualist cultures slightly exceeds those from collectivistic cultures. We can also notice that most enrolled students are 24 and 23 years old, and only very few are in their thirties. Regarding students’ performance (Figs. 2 and 3), we can see that most enrolled students passed the master’s program. However, a considerable amount of failure and dropout needs to be given attention. Moreover, the “Above average” students represent the largest number of students, followed by the “Average” students, then finally the “Below average” students.

### Programming language

This study has been carried out on the Anaconda 4.13.0 (a free OS-independent platform) distribution with Python version 3.8.8. Amongst the Python libraries used in this study are Scikit-learn (for ML algorithms), Pandas (to import and build Data Frames), NumPy (for array computing), and imblearn (for imbalanced data manipulation).

### DM algorithms

Several DM algorithms can be used to predict the students' graduation performance or dropout. However, the literature review suggests that, in general, there is no single DM method that works best in all contexts. Following are the six DM methods that have been applied in this study:

- *Logistic Regression (LR)* A supervised DM algorithm that attempts to distinguish between classes (or categories) by analyzing the relationship between existing independent features (Geng, 2006). In our study, we use the Binary logistic regression in the cases where the dependent feature has only two possible outcomes and Multinomial logistic regression, where the dependent feature has three possible outcomes.
- *Random Forest (RF)* A supervised DM algorithm that builds multiple decision trees and merges them. It uses voting mechanisms from the multiple decision trees to improve the shortcomings of a single DT and get more accurate predictions (Breiman, 2001). Each tree in the random forest provides a class prediction, and the class with the most votes become the model’s prediction.
- *K-Nearest Neighbor (KNN)* A supervised DM algorithm for estimating the likelihood that a data point will become part of one group based on measuring the distance between the classified instance and the closest training examples in the feature space (Clark, 2013).
- *Naïve Bayes (NB)* A supervised DM algorithm that assumes that the features are independent of each other (Harrington, 2011). It is based on the Bayes theorem, which states that if event B has happened, then we can find the probability of event A, and represented as follows: P(A|B) = (P(B|A)* P(A))/P(B).
- *Support Vector Machine (SVM)* A supervised DM algorithm that seeks to find the hyperplane best separating the data points in high dimensional space by maximizing the margin (Clark, 2013).
- *Artificial Neural Networks (ANN)* A series of algorithms that endeavor to recognize underlying relationships in a set of data by mimicking the information process of the human brain (Clark, 2013). It takes place in two phases. First, the network is trained on paired data to determine the input–output mapping. Then, the weights of the connections between neurons are fixed, and the network is used to determine the classifications of a new set of data.

### Evaluation methods

Cross-validation is mainly used in settings where the goal is prediction, and one wants to estimate how accurately a predictive model will perform in practice. Our study evaluates the prediction models using non-exhaustive cross-validation (k-fold cross-validation). In each cross-validation method, we use four evaluation measures which are precision, recall, F1 score, and overall accuracy, explained as follows:

- *Precision*: the ratio of correctly predicted positive observations to the overall predicted positive observations. It is calculated as precision = (TP)/(TP + FP).
- *Recall*: the ratio of correctly predicted positive observations to the total observations in an actual class. It is calculated as recall = (TP)/(TP + FN).
- *F1 score*: the weighted average of Precision and Recall. It is calculated as F1 score = (2 * Precision * Recall)/(Precision + Recall).
- *Accuracy*: the correctness of value, i.e., the ratio of correctly predicted observation to the total observations. It is calculated as accuracy = (TP + TN)/(TP + TN + FP + FN).

where: TP = True positive; FP = False positive; TN = True negative; FN = False-negative.

## Experimental results

In this section of the paper, we present the results obtained from using the six DM algorithms that have been previously described in “DM algorithms” section. For evaluating the performance, and as we have a small dataset, we used tenfold cross-validation (each time, nine of the folds are used for training and one fold is used for testing the model, and the holdout method is repeated ten times).

### Predicting students’ academic achievement

This section provides an overview of the results achieved from using traditional DM algorithms to perform academic predictions. For the predictions performed after the first studying semester, we have selected (1) Gender, (2) Enrollment_age, (3) Culture, (4) Distance, (5) Grade_sem1, (6) F_sem1, (7) Unregistered_exams1, and (8) Registered_exams1 as predictors. For performing the predictions after the second demester, we have selected, (1) Gender, (2) Enrollment_age, (3) Culture, (4) Distance, (5) grade_sem1, (6) grade_sem2, (7) F_semester1, (8) F_semester2, (9) Unregistered_exams1, (10) Unregistered_exams2, (11) Registered_exams1, and (12) Registered_exams2 as predictors.

#### Predict students' academic_status ("Completed" or "Not_completed"):

Table 3 compares the performances of the different DM algorithms that have been used for predicting students’ academic status. We can notice that all the DM algorithms generally provided good accuracy. However, and unsurprisingly, the accuracy is always better when performing the prediction after the second semester. We can also notice that the models best predict the “completed” students (which represents the majority class). For instance, in the case of LR, the precision, recall, and the F1 score reached 0.91, 0.98, and 0.95, respectively, for the “Completed” class. On the other hand, the precision, recall, and F1 score are 0.48, 0.14, and 0.21 for the “Not_completed” class.

**Table 3:** Performance of the different DM algorithms in predicting the completion and non-completion of a degree

DM Algo	Prediction		After the 1st semester				After the 2nd semester			
	Performance measure		Precision	Recall	F1	Accuracy	Precision	Recall	F1	Accuracy
LR	Class	Completed	0.91	0.98	0.95	0.90	0.94	0.95	0.95	0.90
		Not_completed	0.48	0.14	0.21		0.40	0.34	0.37	
RF	Class	Completed	0.93	0.98	0.96	**0.92**	0.95	0.98	0.97	**0.94**
		Not_completed	0.68	0.35	0.46		0.70	0.42	0.53	
KNN (K = 5)	Class	Completed	0.93	0.98	0.95	0.91	0.94	0.98	0.96	0.93
		Not_completed	0.57	0.28	0.38		0.68	0.37	0.48	
NB	Class	Completed	0.95	0.92	0.93	0.88	0.97	0.91	0.94	0.89
		Not_completed	0.41	0.54	0.47		0.41	0.69	0.51	
SVM (poly)	Class	Completed	0.91	0.98	0.94	0.91	0.94	0.99	0.97	0.94
		Not_completed	0.26	0.10	0.14		0.76	0.35	0.48	
ANN	Class	Completed	0.93	0.97	0.95	0.91	0.95	0.99	0.96	0.93
		Not_completed	0.53	0.34	0.41		0.70	0.37	0.48	

Bold values represent the results with best accuracies

#### Predict students’ academic_grade (“Above average”, “Average”, or “Below average”)

Table 4 compares the performances of the different DM algorithms used to predict the academic grade after the first and second studying semesters. Just like the previous cases, the model works best in predicting the majority class, which is the “Above_average” students, in this case, followed by the second major class (“Average”).

**Table 4:** Performance of the different DM methods in predicting students’ academic grade

DM Algo	Prediction		After the 1st semester				After the 2nd semester			
	Performance measure		Precision	Recall	F1	Accuracy	Precision	Recall	F1	Accuracy
LR	Class	Above average	0.83	0.85	0.84	**0.79**	0.85	0.85	0.85	0.80
		Average	0.74	0.79	0.76		0.76	0.79	0.77	
		Below average	0.62	0.22	0.33		0.71	0.49	0.58	
RF	Class	Above average	0.83	0.84	0.84	0.77	0.87	0.85	0.86	**0.81**
		Average	0.71	0.76	0.73		0.75	0.82	0.78	
		Below average	0.60	0.32	0.41		0.67	0.36	0.46	
KNN (K = 5)	Class	Above average	0.80	0.82	0.81	0.74	0.85	0.85	0.85	0.79
		Average	0.68	0.72	0.70		0.74	0.79	0.76	
		Below average	0.57	0.28	0.38		0.64	0.36	0.46	
NB	Class	Above average	0.82	0.86	0.84	0.76	0.82	0.86	0.84	0.78
		Average	0.73	0.69	0.71		0.76	0.68	0.72	
		Below average	0.48	0.44	0.46		0.54	0.69	0.61	
SVM (poly)	Class	Above average	0.79	0.90	0.84	0.76	0.83	0.90	0.86	0.80
		Average	0.73	0.69	0.70		0.78	0.75	0.76	
		Below average	0.40	0.04	0.08		0.69	0.40	0.51	
ANN	Class	Above average	0.81	0.83	0.82	0.75	0.83	0.83	0.83	0.77
		Average	0.69	0.73	.0.71		0.71	0.74	0.73	
		Below average	0.57	0.28	0.38		0.55	0.38	0.45	

Bold values represent the results with best accuracies

### Dealing with imbalanced datasets using SMOTE

By viewing the results in “Predicting Students’ academic achievement” section, one can notice that all the classifiers achieved high accuracy. However, low precession, recall, and F1 score for the minority classes. These misleading results are typical when analyzing imbalanced data. Several techniques have been proposed to solve the problems associated with learning from imbalanced data. Those techniques are (i) resampling (by either oversampling the minority class or under-sampling the majority class), (ii) feature selection, and (iii) cost-sensitive learning. Since we have a limited dataset and a small number of features, over-sampling is the optimal approach. Over-sampling simulates data points to enhance balance across the classes. There are several over-sampling techniques. Our study explores using Synthetic Minority Oversampling Technique (SMOTE), which was proposed to improve random oversampling as it overcomes the overfitting problem posed by random oversampling (Chawla et al., 2002). SMOTE synthesizes new minority instances between existing (real) minority instances. These synthetic training records are generated by selecting one or more of the k-nearest neighbors for each example in the minority class. Then, the data is generated by randomly choosing the features between those two data points. After the oversampling process, the data is reconstructed, and the classification models can be applied to the processed data. Tables 5 and 6 below show the significant improvements in predicting the minority classes after applying SMOTE. For instance, the minority class F1 score using LR raised by 60% after the first semester and 47% after the second semester. As for RF, the F1 score for the minority class raised by 44% and 39% after the first and second semester, respectively. Also, KNN raised by 39% and 36% after the first and second semester, respectively.

**Table 5:** Performance of the DM methods in predicting the completion and non-completion of a degree using SMOTE

DM Algo	Prediction		After the 1st semester				After the 2nd semester			
	Performance measure		Precision	Recall	F1	Accuracy	Precision	Recall	F1	Accuracy
LR	Class	Completed	0.82	0.79	0.81	0.81	0.84	0.84	0.84	0.84
		Not_completed	0.80	0.83	0.81		0.84	0.84	0.84	
RF	Class	Completed	0.92	0.87	0.90	**0.90**	0.93	0.91	0.92	**0.92**
		Not_completed	0.88	0.93	0.90		0.91	0.93	0.92	
KNN (K = 5)	Class	Completed	0.77	0.76	0.77	0.77	0.85	0.83	0.84	0.84
		Not_completed	0.76	0.78	0.77		0.83	0.86	0.84	
NB	Class	Completed	0.82	0.78	0.80	0.81	0.84	0.84	0.84	0.84
		Not_completed	0.79	0.83	0.81		0.84	0.84	0.84	
SVM (poly)	Class	Completed	0.76	0.83	0.79	0.78	0.82	0.87	0.84	0.84
		Not_completed	0.81	0.74	0.77		0.86	0.81	0.83	
ANN	Class	Completed	0.84	0.80	0.82	0.82	0.88	0.80	0.84	0.84
		Not_completed	0.81	0.85	0.83		0.81	0.89	0.85	

Bold values represent the results with best accuracies

**Table 6:** Performance of the different DM methods in predicting the academic grade using SMOTE

DM Algo	Prediction		After the 1st semester				After the 2nd semester			
	Performance measure		Precision	Recall	F1	Accuracy	Precision	Recall	F1	Accuracy
LR	Class	Above average	0.83	0.84	0.83	0.75	0.86	0.84	0.85	0.81
		Average	0.65	0.64	0.65		0.71	0.73	0.72	
		Below average	0.76	0.76	0.76		0.86	0.85	0.85	
RF	Class	Above average	0.83	0.84	0.84	**0.82**	0.89	0.85	0.87	**0.87**
		Average	0.75	0.72	0.74		0.80	0.81	0.81	
		Below average	0.88	0.91	0.89		0.92	0.94	0.93	
KNN (K = 5)	Class	Above average	0.76	0.77	0.77	0.70	0.83	0.80	0.81	0.81
		Average	0.63	0.58	0.60		0.73	0.72	0.73	
		Below average	0.71	0.76	0.74		0.87	0.91	0.89	
NB	Class	Above average	0.81	0.85	0.83	0.74	0.81	0.86	0.84	0.80
		Average	0.63	0.58	0.60		0.71	0.68	0.69	
		Below average	0.77	0.78	0.73		0.87	0.86	0.86	
SVM (poly)	Class	Above average	0.74	0.86	0.80	0.70	0.81	0.87	0.84	0.80
		Average	0.59	0.55	0.57		0.72	0.69	0.70	
		Below average	0.76	0.69	0.72		0.88	0.84	0.86	
ANN	Class	Above average	0.81	0.80	0.80	0.71	0.85	0.80	0.82	0.78
		Average	0.58	0.61	0.59		0.65	0.74	0.69	
		Below average	0.74	0.72	0.73		0.87	0.79	0.83	

Bold values represent the results with best accuracies

### Feature importance on the overall prediction

Feature Importance refers to the techniques that calculate a score to each input feature for a given model where the scores represent the “importance” of each feature. A higher score means that the specific feature will have a more significant effect on the predictive model. There are various functions for generating feature importance. However, since Random Forest provided the best accuracy, it is reasonable to find the impact of each feature on the predictions performed by that classifier. Therefore, we use the Random forest permutation importance measurement, which was introduced by Breiman (2001). The feature selections are performed by looping through each column in the dataset while making predictions, shuffles the column, and making predictions with the shuffled column. If a column is significant to making predictions, shuffling that particular column should increase the error term and vice-versa. Therefore, those columns that lead to a maximum increase in error (loss function) are considered the most important.

#### Feature importance on the predictions performed after the 1st semester

By viewing Table 7, one can notice that the most significant attribute for performing the predictions after the first semester is “Grade_sem1” followed by “Distance”, then “Culture”. Moreover, “Registered_exams1” and “F_sem1” have a minor impact. On the other hand, “Gender”, “Enrollment_age”, “Unregistered_exams1” have the most negligible impact on the prediction.

**Table 7:** Features level of importance on the predictions performed after the 1st semester

Feature	Importance measure	
	Predicting the Academic_status after the 1st semester	predicting the Academic_grade after the 1st semester
Grade_sem1	**0.27**	**0.36**
Distance	0.18	0.14
Culture	0.17	0.12
Registered_exams1	0.10	0.10
F_sem1	0.10	0.09
Enrollment_age	0.08	0.09
Unregistered_exams1	0.05	0.06
Gender	0.05	0.04

Bold values represent the features with most influence on the predictions

#### Feature importance on the predictions performed after the second semester

By viewing Table 8, one can see that the most significant attributes for performing the predictions after the second semester are “Grade_sem2” followed by “Grade_sem1”. While “Culture” and “Distance” have some effect on the prediction, the rest of the features have no significant impact.

**Table 8:** Features level of importance on the predictions performed after the 2nd semester

Feature	Importance measure	
	Predicting the Academic_status after the 2nd semester	predicting the Academic_grade after the 2nd semester
Grade_sem2	**0.30**	**0.33**
Grade_sem1	0.14	0.22
Culture	0.11	0.07
Distance	0.10	0.06
F_sem2	0.08	0.06
Registered_exams2	0.06	0.05
Registered_exams1	0.05	0.05
F_sem1	0.05	0.04
Enrollment_age	0.04	0.04
Unregistered_exams2	0.03	0.03
Unregistered_exams1	0.02	0.03
Gender	0.02	0.02

Bold values represent the features with most influence on the predictions

## Discussion of the results

This section answers our research questions and discusses the results obtained from the predictive models. Our first research question was whether it is possible to accurately predict the academic achievement of master’s students at an early stage. We have built four initial models to answer this research question; two are designed to make predictions after the first studying semester, and two are designed to perform the predictions after the second semester. By going back to Tables 3 and 4, we can notice that the results of predicting the largest classes (“Complete” and “Above_average”) are better than the rest of the classes (“Not_completed”, “Average”, and “Below_average”). This finding was also reported by Nguyen Thai Nghe et al. (2007) which stated that the accuracy of the majority class is higher in their academic prediction study. The minority class is always more challenging to predict because there are only few examples of this class to train on.

**Fig. 4:**
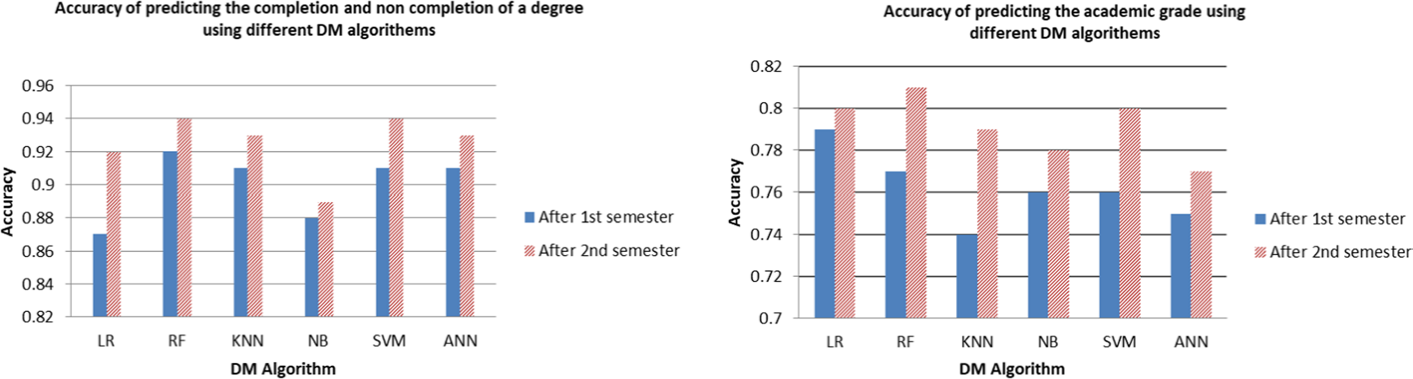
A comparison between the accuracy of the academic predictions performed after the 1st semester and those performed after the 2nd semester

As a general trend, the predictions performed after the second semester yield more significant results than those performed after the first semester (see Fig. 4). In our view, it is reasonable since after the second semester we have a more realistic picture of students' performance than we do at the end of the first semester. We can also notice that predicting the academic status (which comprises two classes) is more accurate than predicting the graduation grade (which comprises three classes). This supports the findings of Nghe et al. (2007), who reported that predicting two-class problems produces more accurate results than predicting three or more class problems (i.e., the more the classes, the more challenging the prediction is). To get into more details regarding the performance of the classifiers, we can see that they gave similar accuracies, with RF performing the best in most cases (0.92- 0.94 in the cases predicting the academic status and 0.77- 0.81 in the cases predicting the graduation grade). This is similar to the results of Zhao et al. (2020) who reported that RF performed the best among seven other algorithms. This is not surprising because RF is an ensemble algorithm that uses bagging as the ensemble method and decision trees as the individual model. Ensemble algorithms can be more accurate than single models as they tend to repeat the process many times such that the model learns the data and makes proper predictions. Another reason behind the excellent performance of RF is that it chooses features randomly during the training process. Therefore, it does not depend highly on any specific set of features. This randomized feature selection is a unique characteristic of RF.

**Fig. 5:**
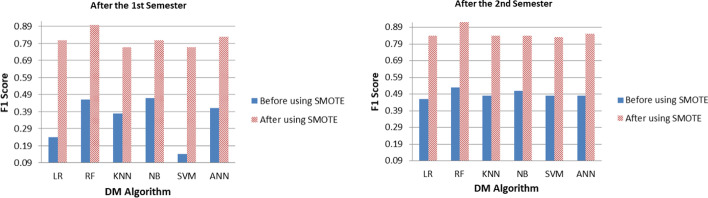
A comparison between the achieved F1 scores for the minority class (Not_completed) using DM methods with and without SMOTE

After briefly discussing the accuracy of the initial models, the question that comes to mind is whether the models are reliable for practical usage. Although we achieved high prediction accuracy in all four models, they are misleading results and unreliable for implementation. That is because other evaluation methods such as the precision, recall, and F1 score for the minority classes are not sufficient enough. We worked on that issue by using SMOTE (Tables 5 and 6). Figure 5 below is an example that compares the F1score of the minority class (Not_completed) before using SMOTE and after using it. Although the accuracy of the classifiers slightly decreased, they are more applicable as we were able to have high precision, recall, and F1 score for the minority classes. We can also notice that in all four models, the RF classifier continues to perform the best compared to the rest of the DM algorithms that have been explored in this study (0.82- 0.92 accuracy).

The second research question in this study is regarding finding out which attributes have the most effect on the prediction of students’ academic achievement. As previous research has shown that personal features, such as marital status, have an impact on student performance, many researchers such as Nghe et al. (2007) and Zhao et al. (2020) examined such features on predicting students’ academic achievements. Moreover, personality features such as motivation and competence, are known to have a strong effect on students’ achievement as proved by Jeno et al. (2018). However, such features are challenging to collect, i.e., it is not common for academic institutes to collect such data during the enrollment process. Therefore, we find that using such features is not effective for generalizing the predictive models and testing them in different academic institutions. In this study, we seek to perform academic predictions using only easy to collect attributes that are available at any university database. Using this approach allows us to test our models across different academic disciplines and facilities.

We have found that the most crucial attributes for performing the predictions after both the first and the second semester are the achieved academic grades in each semester (importance rate: 14–36%). This supports the finding of Rotem et al. (2020) and Asif et al. (2017). The second most important features are culture and distance, with an importance of 7–17% and 6–18%, respectively. Even though the distance between students' accommodations and universities has been a subject of interest for researchers and universities for decades (Simpeh & Akinlolu, 2018), it has not yet become common to use it as a predictor for performing academic predictions. Students who live far from campus are more likely to fail or dropout as it is difficult for them to attend classes on a regular basis. In addition, they are less likely to make use of university facilities (e.g., library) for an extended period of study time.

As discussed in the literature, cultures' behavior toward learning may differ. Generally speaking, students from individualistic cultures tend to have a higher desire to compete with themselves and with others. Therefore, they have higher chances of succeeding the master’s program. However, there are other factors that can influence international students (which are “collectivistic” in our study) to dropout from German educational programs. Those factors include poor linguistic proficiency, financial problems, lack of social and academic integration, and misconceptions regarding the teaching and learning culture at German higher education institutions (Kercher, 2018).

Attrition rates are known to be higher among students who failed courses than among those who did not, and the rates increase as the number of failed courses increase (Ajjawi et al., 2020). In our study, the number of failed courses were surprisingly found to have a minor effect on predicting the academic status and the graduation grade (with an importance rate of 4–10%). This contradicts the findings of Alturki and Alturki (2021) and Kabakchieva (2013), who observed that the number of failed courses is essential for predicting bachelor's students' achievement. This inconsistency may be because course failure in master's programs is not as common as in bachelor's programs.

Balancing the academic load is vital to students' academic achievement (Alturki et al., 2020). In fact, Alemu Yehuala (2015) found that it is one of the main significant attributes for predicting academic achievement. We tested this theory by investigating the impact of the number of registered and unregistered exams per semester. We found that the number of registered exams has a minor effect with a 5–10% importance rate. As for the number of unregistered exams, it has even a lower effect (2–6%) compared to the rest of the post-enrollment features.

Moreover, we found that enrollment age has almost no effect (4–9%). This finding is in line with the findings of Kovačić (2010). However, it contradicts the finding of Zewotir et al. (2015) who observed that age matters in predicting master students time to graduate or dropout. In our case, it does not surprise us that age did not influence the prediction as there is no significant gap between the ages of most applicants (most students are in their twenties). Researchers have conducted studies at different levels throughout the world that have shown a significant difference in academic performance between males and females. Therefore, gender has been used the most in the literature compared to other demographics in predicting academic achievement (Alturki et al., 2020). In our case, gender does not affect the prediction, as it had an importance rate of only 2–4%. This is also in line with the findings of Kovačić (2010), Osmanbegović et al. (2012), and Zewotir et al. (2015).

## Study limitations

Due to time constraints, this study was only performed on one studying program (Business Informatics). However, using the same prediction models in different master’ s programs could give us more insights into whether the predictive models could be generalized and sufficiently work for other programs.

Ensemble methods are known for avoiding overfitting and improving predictions. In this study, we used Random Forests, which is a Bagging (or bootstrap aggregation) method. However, other ensemble methods, such as boosting and stacking, are worth exploring.

Although there are many approaches to dealing with imbalanced datasets, oversampling techniques are optimal for our predictive models. Therefore, we chose to explore SMOTE. However, other oversampling techniques such as adaptive synthetic sampling (ADASYN) and Data augmentation can also be explored.

## Conclusion and future works

Collecting a wide range of student data, other than academic performance, for instance, students' health issues and workload, if employed, can be beneficial for predicting students' academic achievement and reducing students’ dropout. However, the downside is that they are expensive to gather. Therefore, the main objective of this study was to perform academic predictions at a master’ s degree level using only the data that can be easily collected at the beginning of a studying program. We compared the performance of six classifiers, namely: LR, RF, NB, KNN, SVM, and ANN, in predicting students' academic performance and explored using SMOTE to improve our predictions and deal with our imbalanced dataset. Results from our prediction models reveal that it is possible to predict academic achievement with a high accuracy using only a small set of features. Those results can assist in building an early warning system. Such a system will allow instructors to know the students at risk of dropping out or those with higher chances of failure and need to be given special attention. It will help academic institutions increase the academic success and reduce the financial loss caused by students’ dropout or failure. However, in order to benefit the most from such system, it must be carefully constructed and continuously monitored.

Future studies should expand on our study by performing earlier predictions (prior to enrollment) as this can bring more benefits to the educational society. Alturki and Stuckenschmidt, (2021) suggested that earlier predictions can be achieved using pre-enrollment tests. Moreover, we highly endorse testing the predictive modes on other master programs. Also, future studies should explore more ensemble techniques to perform academic predictions. We also suggest investigating other oversampling techniques to deal with the imbalanced datasets. Finally, we highly encourage educators and researchers to apply more EDM studies to postgraduate students to have a more realistic comparison between undergraduate and postgraduate EDM studies.

## Data Availability

More data is available from the corresponding author on reasonable request.

## Abbreviations

DAAD: Deutsche Akademische Austauschdienst
DM: Data mining
ECTS: European credit transfer system
EDM: Educational data mining
ANN: Artificial neural networks
KNN: K-nearest neighbor
LR: Logistic regression
RF: Random forest
SMOTE: Synthetic minority oversampling technique
SVM: Support vector machine
NB: Naïve Bayes
